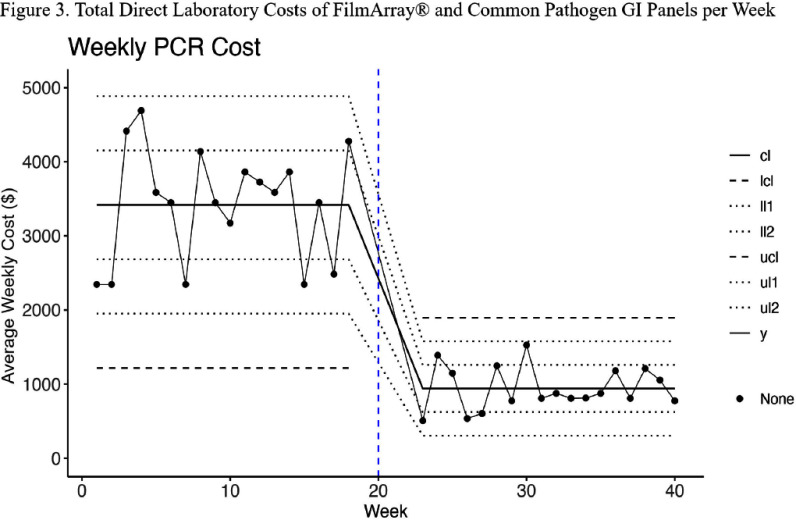# Diagnostic Stewardship of Gastrointestinal Pathogen Panels: Impact on Test Utilization and Hospital Costs

**DOI:** 10.1017/ash.2025.309

**Published:** 2025-09-24

**Authors:** Aaron Pathak, Todd Lasco, Mayar Al Mohajer, Rogers Kisame

**Affiliations:** 1 Baylor College of Medicine; 2 Baylor College of Medicine; 3 Baylor College of Medicine

## Abstract

**Background:** While broad gastrointestinal (GI) multiplex polymerase chain reaction (PCR) panels can test for various bacterial, viral, and parasitic pathogens, their overuse may yield a high financial burden on hospital systems without clear clinical relevance of all covered organisms. This study aims to assess whether a multifaceted quality improvement intervention directing clinicians to a more limited panel and requiring several restriction criteria would reduce direct hospital costs for patients with suspected infectious diarrhea. **Methods:** Our quasi-experimental study included patients from a quaternary academic medical center in Texas. In the pre-intervention period (March 2024-June 2024), the Biofire® FilmArray® Gastrointestinal Panel (BioFire Diagnostics, Salt Lake City, UT) was the preferred test for patients presenting with suspected infectious diarrhea and had minimal ordering restrictions (Figure 1). In the post-intervention period (August 2024- November 2024), a second narrower panel (GI Common Pathogen PCR panel) was introduced as the preferred test with some restrictions, while the Biofire® FilmArray® GI Panel was only available to severely immunosuppressed patients and required Infectious Diseases consultation. The restriction criteria were built in the Epic electronic health system (Epic System Corporation, Verona, WI). Information on the intervention was distributed through email memorandums and an internal secure clinical messaging platform. Count control charts were used to visualize the number of FilmArray® GI Panels conducted, while individual control charts were used for the direct laboratory costs of both GI panels. **Results:** 893 patients had suspected infectious diarrhea in the study period (451 pre-intervention, 442 post-intervention). The average number of weekly FilmArray® GI Panel tests performed dropped from 24.8 to 1.9 (Figure 2), and an average of 21.9 GI Common Panel tests per week were performed in the post-intervention period. The average weekly testing cost decreased from $3,418.10 to $940.40 after the intervention (Figure 3). The two control charts demonstrated the presence of special cause variation for both outcomes (weekly FilmArray® GI Panel tests and combined costs), indicating a change after the intervention. **Conclusion:** Although the total number of tests did not change after adjusting the restriction criteria, this intervention significantly reduced the direct laboratory costs of the GI Panels after guiding clinicians to a more economical test (GI Common Panel), with an estimated annual savings of $128,840. This study provides a diagnostic stewardship opportunity for cost reduction in healthcare systems. Future evaluation will analyze its impact on antimicrobial utilization and infection control metrics.

**Figure:**
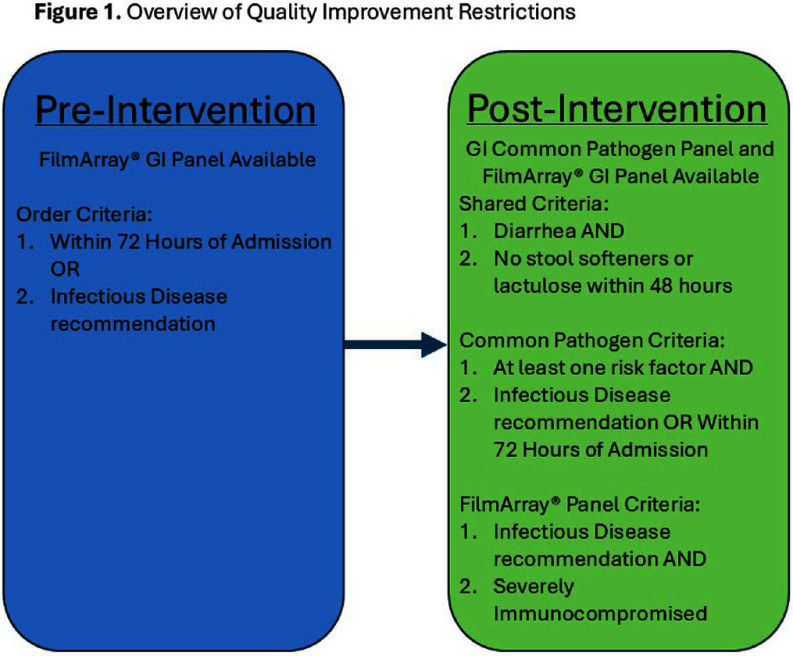

**Figure:**
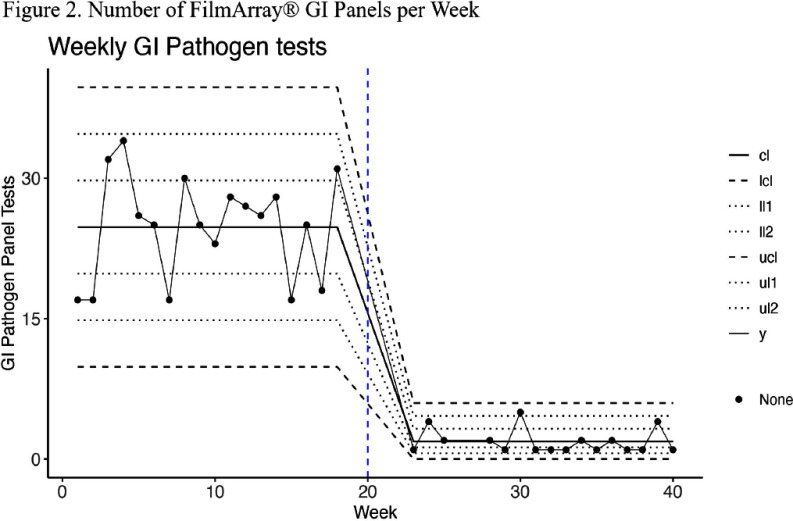

**Figure:**